# Supplementary material for: Photodegradable Hydrogels for On‐Demand Modeling of Age‐Related Spatiotemporal ECM Deformation
Source: Small. 2026 Feb 20;22(27):e09246. doi: 10.1002/smll.202509246 (PMC13173314; doi:10.1002/smll.202509246)
Supplement: Supplementary file 1 — Supporting File: smll72894‐sup‐0001‐SuppMat.docx. [file SMLL-22-e09246-s001.docx]

## Supporting Information


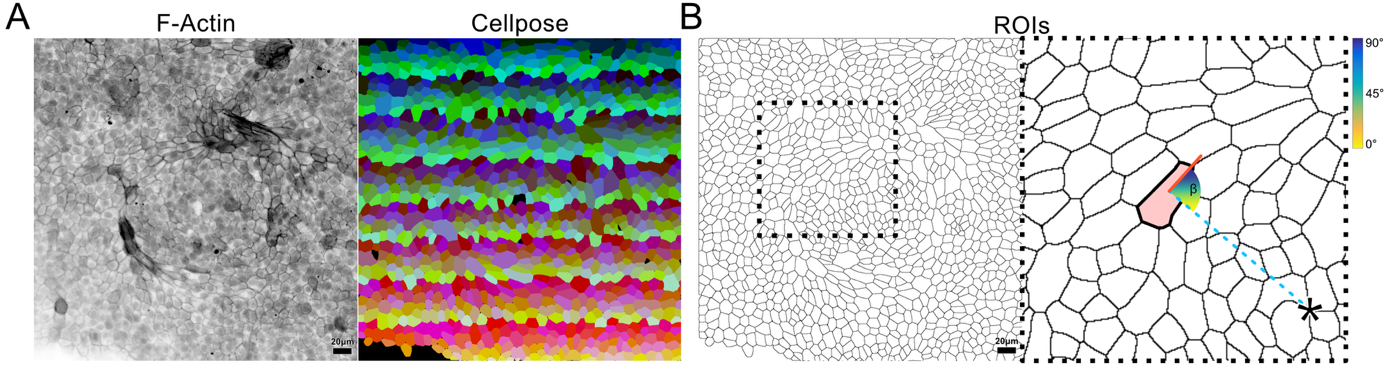


**Supplementary Figure 1: Methodology for quantifying cellular angles to the origin. A)** Representative maximum intensity projection of F-actin-stained RPE with the Cellpose segmentation output picture. **B)** Representative maximum projection of a ZO-1-stained RPE with the zoomed-in region highlighted by a dashed line. The center of the artificial drusen is marked by an asterisk. The absolute angle of the cell is indicated by the red solid line, while the vector from the center of the highlighted cell to the center of the artificial drusen is indicated by the dotted line. The scalar product between the two vectors is indicated by angle β and lies between 0° and 90°, as shown by a color gradient from yellow to blue.


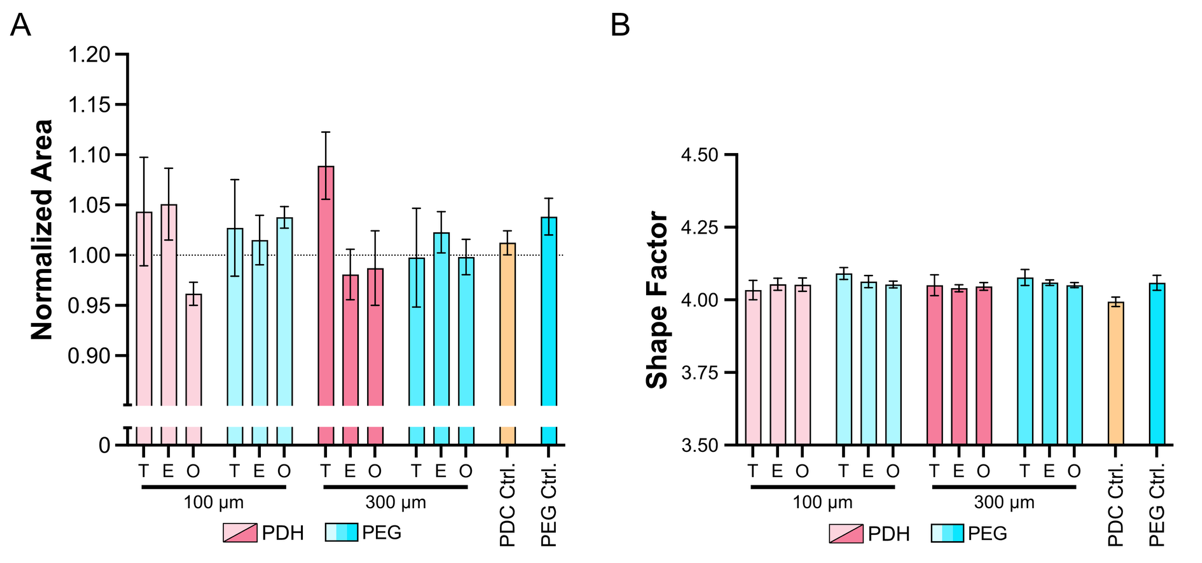


**Supplementary Figure 2: RPE cell areas and shape factors. A)** Bar plot showing normalized average cell area ± SEM in all conditions. **C)** Bar plot showing the normalized shape factor ± SEM in all conditions**.**


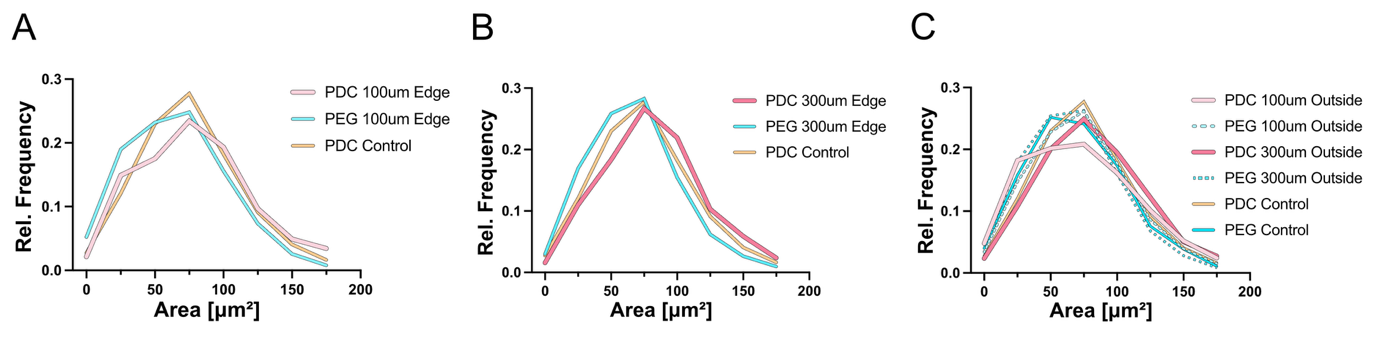


**Supplementary Figure 3: Cell area distribution.** Relative frequency distributions of the cell areas for RPE on 100 µm Edge (A) and controls, 300 µm Edge and controls (B), and 100 µm, 300 µm Outside and controls (C).

**Supplementary Table 1**

| Number of technical replicates per independent replicate | | | |  |  |  |  |  |
| --- | --- | --- | --- | --- | --- | --- | --- | --- |
|  |  | | Independent replicates (N) | | | | | |
| Experiment Name | | Condition | 1 | 2 | 3 | 4 | 5 | 6 |
| Additive Illumination | 100 and 300µm PDH | | 6 | 4 | 5 |  |  |  |
| Absolute swelling height | 100 and 300µm PDH | | 4 | 8 | 3 |  |  |  |
| Hydrogel and artificial drusen stiffness | PDH | | 42 | 11 | 43 |  |  |  |
|  | 100µm PDH | | 3 | 10 | 8 |  |  |  |
|  | 300µm PDH | | 4 | 11 | 13 |  |  |  |
|  | PEG | | 32 | 136 | 4 |  |  |  |
| Swelling height with cells | 100µm | | 6 | 6 | 5 | 5 |  |  |
|  | 300µm | | 4 | 3 | 6 | 5 |  |  |
| Monolayer height | 100µm PDC | | 1443 | 1443 | 1443 | 1443 | 1443 |  |
|  | 300µm PDC | | 1443 | 1443 | 1441 |  |  |  |
|  | 100µm PEG | | 1443 | 1443 | 1443 | 1443 |  |  |
|  | 300µm PEG | | 1443 | 1443 | 1443 |  |  |  |
|  | PDC Background | | 1443 | 1443 | 1438 | 1443 | 1577 |  |
|  | PEG Background | | 1443 | 1443 | 1443 |  |  |  |
| Relative frequency distribution of area | 100µm PDC | | 3933 | 3109 | 1469 | 2847 | 2438 |  |
|  | 300µm PDC | | 4248 | 3083 | 1303 | 3373 |  |  |
|  | 100µm PEG | | 3657 | 4606 | 3398 | 4645 | 2695 | 4179 |
|  | 300µm PEG | | 4248 | 4670 | 3675 | 2768 | 4284 |  |
|  | PDC Background | | 3320 | 2867 | 359 | 2280 | 5210 | 1072 |
|  | PEG Background | | 4384 | 3261 | 4573 |  |  |  |
| Angle to origin | 100µm PDC | | 2438 | 3109 | 2847 |  |  |  |
|  | 300µm PDC | | 3083 | 3373 | 1303 |  |  |  |
|  | 100µm PEG | | 4645 | 4606 | 3658 | 3397 | 2695 | 4179 |
|  | 300µm PEG | | 4670 | 4248 | 3675 | 2768 | 4184 |  |
|  | PDC Background | | 6449 | 3004 | 2280 | 1542 |  |  |
|  | PEG Background | | 4573 | 4384 | 3257 |  |  |  |
| Delta mean traction | 100µm PDC | | 3 | 3 | 3 |  |  |  |
|  | 300µm PDC | | 3 | 3 | 3 |  |  |  |
| Median traction | 100µm PDC | | 3 | 5 | 5 |  |  |  |
|  | 300µm PDC | | 3 | 5 | 5 |  |  |  |
